# Tell Me Your Superpower!

**DOI:** 10.1210/endocr/bqaa021

**Published:** 2020-02-22

**Authors:** Teresa K Woodruff

**Affiliations:** Department of Obstetrics and Gynecology, Northwestern University, Chicago, Illinois

When my nephew, Ryan, was about 8, his favorite question was “What is your superpower?” We would spend hours riding bikes sorting out whether invisibility, shapeshifting, X-ray vision, or teleporting were the best asset. This is the same nephew who joined my lab every summer beginning at age 5 drawing ovarian follicles, which culminated in a Woodruff and Woodruff publication based on hundreds of hours of follicle counting and categorization (1). My lab was amazed that he could work on the microscope for 6 and 7 hours every day, and he kept his notebook open even as we were driving home. Ryan’s superpowers are many and I think a DC comic character named Ryan with an “attention to detail” superpower needs to be invented—this is a strength that is on par with shape-shifting and teleporting!

In my opinion, humans have 3 true superpowers—the ability to see and plan for the future, the ability to invent or create, and the ability to leap. All 3 can be used for good or evil, just as the comic books describe. Let me give you a few examples of the super-secret buttons that can activate these positive attributes in your careers.

First is the ability to see and plan for the future. My grandparents were all farmers, and I remember thinking about how they would plant winter wheat in the fall knowing that a harvest was coming after the long, bleak winter. Animals certainly gather seeds and nuts to store for the winter, but only humans are able to generate plans that allow for sustained, intentional harvests. The ability to think about the future is what drives our interest in religion and questions about any afterlife. We plan for the future in many ways, we create grant plans, we have 18-month calendars to schedule future travel, and we think about retirement. Graduate school is a place where we need this superpower the most, and the villainous side of our superpower is the despair graduate students can feel during the long days and nights when that future self feels unassured (2). Undergraduates come to the university with a date on their back—Class of 2024. Graduate students come to the university without the firm knowledge of what year they will achieve the goal of a PhD. The ability to see and plan for the future can stall and feel out of our hands. It is an inability to trust that the future self is out there which leaves us looking for ways to teleport into a new time–space reality.

Yet that uniquely human ability to think and plan for the future means that, even while in grad school, imagining oneself as successful, rather than questioning whether success is possible, is a good start. I’ve talked about the dailiness of science (my latitude and longitude analogy (3)), and in a scientific career, you don’t need a Marvel comic option, you just need to activate the uniquely human power of planning for success. I keep a weekly “joy jar” that is filled with notes to myself. Every Friday I write one that identifies something good that happened that week. When I look back over the last year, I immediately see the power of time. A joy jar is not a cape, but it is a tool to open yourself up to the dailiness of time and progress and can give hope for your future self.

The second superpower humans possess is the ability to invent or create. This superpower is what we celebrate in each issue of *Endocrinology*, and a few great parts of our knowledge web can be found in the papers referenced here (4–7). I believe that the lab meeting is the best button can you push to release a spider’s web of insight leading to that next invention or creation. Of course, the invent/create superpowers also make humans amazing supervillains in our ability to create waste. All organisms metabolize and create waste. Humans create not only metabolic waste but an incredible amount of manufactured waste. Our planet is almost swimming in the excretions of our superpowers. Scientists and our science must be aware of our role in this problem. I love the many sustainability initiatives that have been launched by universities to make labs more aware of the way we can reduce our lab waste stream.

And now we get to the best superhero feat of all—the ability to leap tall buildings in a single bound! I believe humans are not only uniquely able to see ourselves in the future and therefore create and invent ways to shape our lives and our environment, we are also uniquely able to take gigantic leaps. No other life form on the planet would catapult members of its species out of their native environment into the depths of the sea, the deep recesses of the earth’s crust, or outer space. Humans can leap. Imagination and science are our super-secret weapons. The pages of our journal are filled with the evidence of this power. See especially the new articles on FSH collected under the “Companion Articles” right sidebar in Djurdjica Coss’s mini-review assessing the collection (8).

My own superpower is juggling. My life includes a number of jobs that are all running in parallel—dean of the graduate school, director of my laboratory and grants, head of the Center for Reproductive Science, the Women’s Health Research Institute, the Oncofertility Consortium, and Editor-in-chief of this journal. Folks ask me how I manage all these portfolios. My best answer is that each role is a ball that I’m juggling. You have to know which items are ping pong balls that can fall occasionally and then bounce back up so you can catch them and return them to upward motion, and which are glass balls that can’t be dropped.

You each have superpowers that are unique and those that bind us together as a species. So, what’s left to say but…Engage!
